# NCR as a biomarker for nutritional status and inflammation in predicting outcomes in patients with cancer cachexia: a prospective, multicenter study

**DOI:** 10.1186/s12885-025-13919-1

**Published:** 2025-03-25

**Authors:** Xiangrui Li, Li Deng, Hailun Xie, Shuqun Li, Hong Zhao, Tong Liu, Xiaoyue Liu, Shiqi Lin, ChengAn Liu, Han-Ping Shi

**Affiliations:** 1 Department of Gastrointestinal Surgery/Department of Clinical Nutrition, Beijing Shijitan Hospital, Capital Medical University, Beijing, 100038 China; 2 Beijing International Science and Technology Cooperation Base for Cancer Metabolism and Nutrition, Beijing, 100038 China; 3 Key Laboratory of Cancer FSMP for State Market Regulation, Beijing, 100038 China

**Keywords:** NCR, Systemic inflammation, Malnutrition, Cancer cachexia, Overall survival

## Abstract

**Background:**

Systemic inflammation and nutritional status are key factors affecting the prognosis of patients with cancer cachexia. This study aims to evaluate the prognostic value of a new nutritional and inflammatory index, Prognostic Nutritional CRP Ratio (NCR), in patients with cancer cachexia.

**Methods:**

This prospective multicenter study analyzed 3,447 patients diagnosed with cancer cachexia across over 40 clinical centers in China, from June 2012 to December 2023. The NCR was calculated as BMI × albumin / CRP. The Cox proportional hazards regression model was utilized to analyze hazard ratios (HRs) for all-cause mortality. The relationship between NCR and all-cause mortality was assessed using restricted cubic spline modeling. The optimal cutoff value for NCR was determined through maximally selected rank statistics.

**Results:**

Among the 3,447 individuals diagnosed with cancer cachexia in our study, 2,296 (66.6%) were men, and 1,151 (33.4%) were women. With a median follow-up duration of 45.33 months, the mean age of the participants was 63.8 ± 11.4 years. We observed that lower NCR levels were prevalent among cachexia patients across a spectrum of cancer types, including lung, colorectal, liver, esophageal, breast, ovarian, and cervical cancers. We observed that lower NCR levels were prevalent among cachexia patients across a spectrum of cancer types, including lung, colorectal, liver, esophageal, breast, ovarian, and cervical cancers. This correlation held true across diverse patient subgroups, delineated by gender, age, smoking status, BMI, TNM stage, and tumor types, underscoring the broad applicability of NCR as a prognostic marker. Moreover, our findings highlighted that cancer cachexia patients with higher NCR levels experienced a significantly improved quality of life.

**Conclusion:**

The NCR, indicative of nutritional status and inflammation, is associated with reduced all-cause mortality and could be a valuable prognostic marker for patients with cancer cachexia.

**Supplementary Information:**

The online version contains supplementary material available at 10.1186/s12885-025-13919-1.

## Introduction

Cancer cachexia, a multifaceted syndrome, manifests as a progressive depletion of skeletal muscle mass, optionally accompanied by fat loss, that traditional nutritional interventions fail to counteract, leading to a decline in functional capabilities [1, 2]. Predominantly associated with advanced-stage cancers, it afflicts approximately 50–80% of those diagnosed with cancer, profoundly diminishing their quality of life, treatment efficacy, and overall survival (OS) [3–5]. Identifying sensitive and specific biological markers for predicting the prognosis of patients with cancer cachexia is urgently needed to improve patient treatment and outcomes.

The pathophysiological mechanisms underlying cancer cachexia are intricate, involving changes in the patient's inflammatory status and nutritional state. Inflammation, a pivotal component of the tumor microenvironment, is not merely a hallmark of cancer but also plays a critical role in driving cachexia. Pro-inflammatory cytokines, notably tumor necrosis factor-alpha (TNF-α), interleukin-6 (IL-6), and interleukin-1 beta (IL-1β), significantly contribute to cachexia pathophysiology by promoting muscle wasting and fat loss [6]. T The systemic inflammatory response observed in patients with cancer cachexia, characterized by alterations in peripheral blood cells and inflammatory proteins such as the neutrophil-to-lymphocyte ratio (NLR), platelet-to-lymphocyte ratio (PLR), lymphocyte–C-reactive protein (CRP) ratio (LCR), and inflammatory burden index (IBI), has been independently associated with prognosis [7–9]. Moreover, systemic inflammation adversely affects nutritional well-being, leading to decreased appetite, altered taste perceptions, and an increased metabolic rate, culminating in an energy deficit. This negative energy balance further exacerbates muscle and fat loss, locking patients in a vicious cycle of cachexia progression [10]. Nutritional Status (CONUT) score, Nutritional Risk Index (NRI), and albumin-to-globulin ratio (AGR), have emerged as potential indicators of cancer cachexia and predictors of clinical prognosis [11–13]. These markers provide a quantifiable measure of the impact of cachexia on patients' nutritional health, offering a pathway to tailor nutritional and therapeutic interventions more effectively. The intertwined relationship between systemic inflammation and nutritional status underscores their significant impact on patients with cancer cachexia, crucially influencing survival prognosis. Thus, the accurate assessment of inflammation levels and nutritional status is paramount in prognosis analysis, enabling the development of targeted strategies to mitigate the debilitating effects of cachexia and improve patient outcomes.

This study introduces a novel nutritional and inflammatory index—the Prognostic Nutritional CRP Ratio (NCR), calculated as BMI × albumin / CRP. Previous research has demonstrated that NCR effectively categorizes patients with stage II and III colon cancer, as defined by the Union for International Cancer Control (UICC), for targeted adjuvant therapy [14]. However, the prognostic potential of NCR for patients with cancer cachexia remains unexplored. Our objective is to delineate the relationship between NCR levels and survival outcomes, as well as the quality of life in patients suffering from cancer cachexia. The insights gained from this study could significantly impact the management and treatment of cancer cachexia, highlighting the critical role of early detection and intervention in improving patient care.

## Patients and methods

### Study participants

**Fig. 1:**
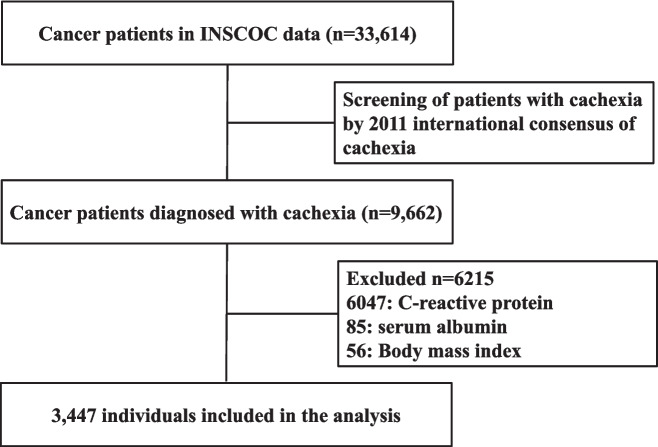
Flow chart of the study design

This project prospectively recruited patients hospitalized at more than 40 clinical centers in China from June 1, 2012, to December 31, 2023. Cases of multiple admissions were each treated as a single entry, with patients providing written consent for participation. Eligible patients met the following criteria: aged 18 years or older, hospitalized for more than 48 h, diagnosed with cancer pathologically, able to independently complete the questionnaire, and without acute infections or severe malnutrition. Data were excluded in instances of incomplete information regarding body mass index (BMI), C-reactive protein (CRP), serum albumin, or duration of survival, leading to the inclusion of 3,447 patients diagnosed with cancer cachexia in the final analysis of the study. (Fig. 1). The participants' mean age was 63.8 ± 11.4 years, with women comprising 33.4% (1,151 out of 3,447) of the cohort. The study was conducted in strict accordance with the ethical principles outlined in the Declaration of Helsinki and received approval from the ethical review boards of all participating centers.

### Data collection and definitions

Cancer diagnoses in the study were confirmed through histological analysis following surgical procedures or needle biopsies. At diagnosis, comprehensive baseline data were collected, encompassing demographic and clinical characteristics such as age, sex, BMI, existing comorbidities (including diabetes mellitus and hypertension), smoking and alcohol consumption habits, tea drinking habits, type of cancer, TNM stage according to the 8th edition of the Union for International Cancer Control (UICC) TNM classification, family history of cancer, treatment approaches, scores from the Patient-Generated Subjective Global Assessment (PG-SGA), scores of the Karnofsky Performance Status (KPS), responses to the European Organization for Research and Treatment of Cancer Quality of Life (EORTC QLQ-C30) Questionnaire, and details of nutritional interventions. Biochemical parameters such as serum albumin, lymphocyte–C-reactive protein (CRP)levels, alanine and aspartate aminotransferase levels (AST and ALT), counts of white blood cells (WBC), neutrophils, lymphocytes, red blood cells (RBC), and platelets were assessed pre-treatment during the initial visit. Prognostic Nutritional CRP Ratio (NCR) we introduced was derived by BMI × albumin to CRP ratio. Body Mass Index (BMI) was determined using the formula: BMI (kg/m^2^) = weight (kg) / (height (m)^2^). The EORTC QLQ-C30 questionnaire, consisting of 30 questions, was used to evaluate the global health status, with subscales assessing symptoms like dyspnea, insomnia, appetite loss, constipation, diarrhea, and financial difficulties, as well as functional aspects including physical, role, social, emotional, and cognitive functions, alongside symptom scales for fatigue, pain, nausea, and vomiting [15]. The standard score (0–100) of each domain was calculated according to the formulae for the EORTC QLQ-C30 [16]: Total score = (physical functioning + role functioning + social functioning + emotional functioning + cognitive functioning + [100 – fatigue] + [100 – pain] + [100—nausea and vomiting] + [100 – dyspnea] + [100 – insomnia] + [100—appetite loss] + [100 – constipation] + [100 – diarrhea])/13. A higher score on the functional and global health status scales indicates a better level of functioning. Conversely, on the symptoms scale, higher scores indicate more severe symptoms, reflecting a negative scoring system.

### Definition of cachexia

The criteria [1] for diagnosing cancer cachexia weight loss > 5% over the previous 6 months (in the absence of simple starvation) or weight loss > 2% in individuals already underweight according to current BMI (< 20 kg/m^2^) or skeletal muscle mass (sarcopenia). Skeletal muscle depletion was assessed based on mid-upper arm muscle area according to anthropometry (men < 32 cm^2^, women < 18 cm^2^). A diagnosis of cancer cachexia is confirmed if any of these conditions are fulfilled.

### Statistical analyses

Overall survival (OS) was defined as the time from the diagnosis of cancer cachexia to the earliest of the following: date of death, withdrawal from the study, end of follow-up (September 30, 2023), or last known contact. The distribution of continuous variables was tested using the Kolmogorov–Smirnov test, and results are reported as mean ± standard deviation for normally distributed data or median with interquartile range (IQR) for data not normally distributed. Prior to analysis, NCR levels underwent logarithmic transformation. Survival analysis, both univariate and multivariate, was conducted using the Cox proportional hazards model, focusing on OS. We calculated hazard ratios (HRs) with 95% confidence intervals (CIs) and presented these findings. We utilized time-dependent area under the curve (AUC) analysis to assess the predictive accuracy of various biomarkers, including BMI, CRP, albumin, and the NCR, over a timeline from 10 to 80 months. The relationship between NCR and nutritional indicators was assessed using Spearman's rank correlation coefficients. The potential non-linear association between NCR levels and HRs was examined through restricted cubic spline regression. An optimal NCR threshold of 309.07 was determined for maximizing the log-rank statistics. Survival outcomes were estimated using Kaplan–Meier curves and assessed by the log-rank test. Covariate subgroup analyses were performed using separate Cox models, stratified by identified covariates. Trends were tested using the Wald test, with quartiles represented by their median values. Interactions were evaluated through multilevel interaction terms in the multivariate models, with significant interactions (*p* < 0.05) depicted in Kaplan–Meier plots for the respective subgroups. All statistical analyses were executed in R software (Version 4.0.2), ensuring rigorous data examination for publication.

## Result

### Characteristics of NCR in patients with cancer cachexia

**Fig. 2:**
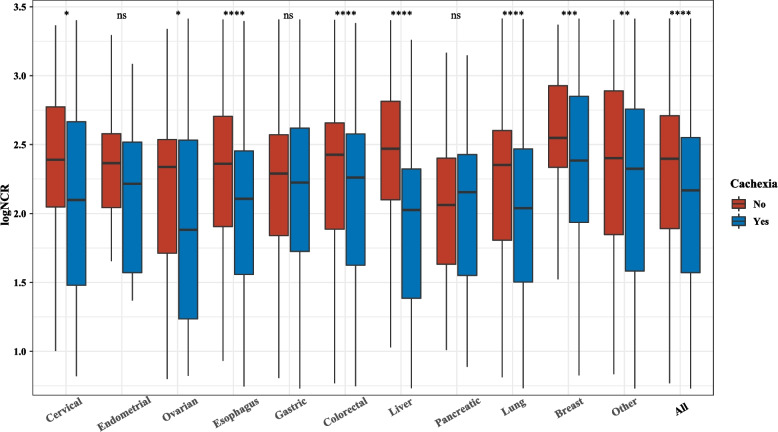
NCR (log transformation) in different cancer types categorized by whether patients had cachexia. ns *p* > 0.05, *p < 0.05, **p < 0.01, ***p < 0.001,

In the presented study involving 33,614 cancer patients, 9,662 were diagnosed with cancer cachexia. Out of these, 3,447 cachexia patients were included in the final analysis (Fig. 1). The median follow-up period was 45.33 months, during which there were 2,066 deaths. We employed the NCR to predict survival in patients with cancer cachexia. The area under the curve (AUC) of the NCR tended to be higher than that of its individual indicators (Supplementary Fig. 1). The association between NCR levels and TNM stages was depicted in Supplementary Fig. 2, showing lower NCR levels in more advanced stages (III and IV). Patients were compared, both with and without cachexia, across different cancer types and stages to evaluate their NCR. Cachexia patients exhibited significantly lower NCR values compared to those without cachexia (*p* < 0.05). Notably, the NCR was markedly lower in cachexia patients with lung, colorectal, liver, esophageal, breast, ovarian, and cervical cancers (Fig. 2). Additionally, a Spearman rank correlation test assessed the relationships between NCR and variables such as age, KPS, PG-SGA, and EORTC QLQ-C30. No correlation was found between NCR and age. The EORTC QLQ-C30 and PG-SGA displayed a strong negative correlation with NCR (men: *R* = −0.27; women: *R* = −0.20; for PG-SGA, men: *R* = −0.31; women: *R* = −0.29), while KPS demonstrated a positive correlation with NCR (men: *R* = 0.27; women: *R* = 0.25) (Supplementary Fig. 3).

### Association between NCR and OS

**Fig. 3:**
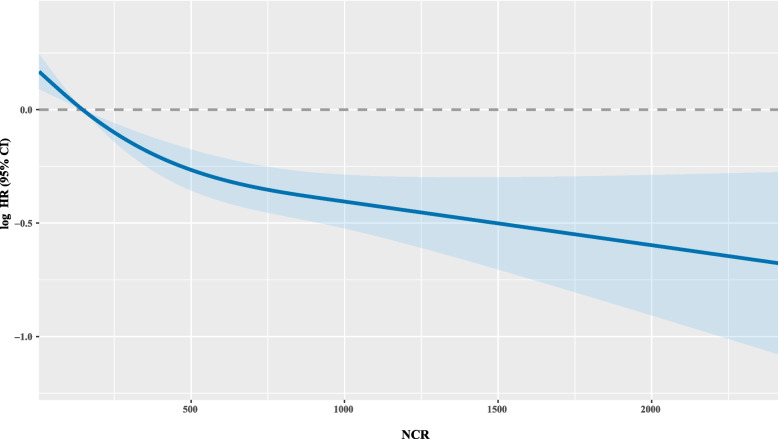
Association between NCR (continuous) and overall survival. The spline was adjusted by sex, age, smoking, tumor type, TNM stage, chemotherapy, surgery, albumin, TBIL, neutrophil count, platelet count, KPS score, PG-SGA score and EORTC QLQ-C30 score. KPS, Karnofsky Performance Status Scale; PG-SGA, patient-generated subjective nutrition assessment; EORTC QLQ-C30, European Organization for Research and Treatment of Cancer Quality of Life Questionnaire; CI, confidence interval; HR, hazard ratio

We conducted univariate and multivariate Cox regression analyses to evaluate the association between NCR levels and OS in patients with cancer cachexia (see Supplementary Table 1). The univariate analyses revealed that most baseline characteristics were associated with an increased risk of mortality. However, a higher NCR was linked to a reduced risk of mortality (HR 0.66, 95% CI: 0.61–0.71). In the multivariate analysis, factors such as smoking, tumor type, TNM stage, chemotherapy, surgery, albumin levels, NCR, TBIL, neutrophil and platelet counts, and the PG-SGA score were identified as independent prognostic factors for survival. The cutoff value for NCR associated with OS was established at 309.07 (Supplementary Fig. 4A). Further analysis through restricted cubic spline modeling demonstrated a negative correlation between the risk of mortality and NCR levels (Fig. 3). Additionally, NCR was associated with a positive prognosis in patients with cancer cachexia after adjusting for sex, age, tumor type, TNM stage, smoking, chemotherapy, surgery, TBIL, neutrophil count, platelet count, and EORTC QLQ-C30 score (Table 1). Moreover, each 1 standard deviation (SD) increase in NCR was associated with a 22% and 21% reduction in mortality risk in models B and C, respectively (HR 0.78, 95% CI 0.73–0.84, *p* < 0.001; HR 0.79, 95% CI 0.74–0.86, *p* < 0.001). When NCR was segmented into quartiles, the third (155.93–341.76) and fourth quartiles (> 341.76) were positively correlated with an improved prognosis compared to the first quartile, indicating a better prognosis (*P for trend* < 0.001).

**Table 1:** The association between NCR and hazard ratio of cancer patients with cachexia

NCR	Model a		Model b		Model c	
	HR 95%CI	*p*-value	HR 95%CI	*p*-value	HR 95%CI	*p*-value
Continuous (per SD)	0.74 (0.68,0.79)	< 0.001	0.78 (0.73,0.84)	< 0.001	0.79 (0.74,0.86)	< 0.001
Cut-off value						
Low (< 309.07)			ref		ref	
High (≥ 309.07)	0.49 0.43,0.56)	< 0.001	0.55 (0.48,0.63)	< 0.001	0.56 (0.49,0.64)	< 0.001
Interquartile						
Q1 (< 39.49)			ref		ref	
Q2 (39.49–155.93)	0.91 (0.79,1.05)	0.194	0.95 (0.82,1.09)	0.473	0.96 (0.83,1.10)	0.574
Q3 (155.93–341.76)	0.73 (0.63,0.85)	< 0.001	0.82 (0.70,0.95)	0.010	0.81 (0.70,0.94)	0.007
Q4 (≥ 341.76)	0.45 (0.38,0.52)	< 0.001	0.53 (0.45,0.62)	< 0.001	0.54 (0.46,0.63)	< 0.001
* p* for trend		< 0.001		< 0.001		< 0.001

Model a: No adjusted

Model b: Adjusted by age, sex, TNM stage

Model c: Adjusted by age, sex, TNM stage, tumor type, surgery, radiotherapy, chemotherapy, hypertension, diabetes, smoking, drinking, family history

### Demographics and disease traits stratified by NCR in cancer cachexia patients

Patients with cancer cachexia were divided into two groups based on the NCR cutoff: a low NCR group comprising 2,462 individuals and a high NCR group with 985 individuals (Table 2). Kaplan–Meier curves and log-rank tests showed that the high NCR group had a significantly better prognosis, as illustrated in Supplementary Fig. 4B. A comparison of demographic and clinical characteristics between the groups revealed that high NCR was associated with younger age, higher BMI, specific tumor types such as lung cancer, lower TNM stages, and receipt of radiotherapy and surgery. Additionally, patients in the high NCR group had lower CRP and TBIL levels, higher albumin and red blood cell counts, and lower neutrophil and platelet counts. These patients also scored higher on the KPS scale, and had lower scores on the PG-SGA and the EORTC QLQ-C30 scales (Table 2). Further analysis indicated that high NCR correlated with improved OS across most tumor types, except for liver and pancreatic cancers, where no significant relationship with OS was observed (Supplementary Table 2). Moreover, patients with a high NCR reported a better quality of life in terms of functional and global health status, as well as lower symptom severity according to the EORTC QLQ-C30 (Supplementary Table 3), highlighting the positive impact of high NCR on the quality of life in patients with cancer cachexia.

**Table 2:** Demographics and clinicopathologic characteristics of patients with cancer cachexia stratified by NCR

Characteristic	NCR low *n* = 2462	NCR high *n* = 985	*p*-value
Population Characteristic			
Sex (%)			
Male	1643 (66.7)	653 (66.3)	0.836
Female	819 (33.3)	332 (33.7)	
Age (mean (SD))	66.34 (11.35)	64.26 (11.57)	< 0.001
BMI, kg/m^2^, mean (SD)	21.09 (3.13)	22.16 (3.25)	< 0.001
Diabetes, yes, n (%)	245 (9.9)	80 (8.1)	0.110
Hypertension, yes, n (%)	509 (20.7)	175 (17.8)	0.059
Smoke, yes, n (%)	1259 (51.1)	468 (47.5)	0.059
Alcohol, yes, n (%)	652 (26.5)	230 (23.4)	0.063
Tea, yes, n (%)	595 (24.2)	221 (22.4)	0.300
Clinical Characteristic			
Lung cancer (%)	651 (26.4)	191 (19.4)	< 0.001
Gastric cancer (%)	599 (24.3)	265 (26.9)	0.126
Colorectal cancer (%)	503 (20.4)	219 (22.2)	0.259
Esophagus cancer (%)	220 (8.9)	50 (5.1)	< 0.001
Pancreatic cancer (%)	88 (3.6)	14 (1.4)	0.001
Liver cancer (%)	74 (3.0)	21 (2.1)	0.193
Gynecological and breast cancer (%)	145 (5.9)	86 (8.7)	0.003
Other cancer (%)	182 (7.4)	139 (14.1)	< 0.001
TNM stage (%)			
I	190 (7.7)	121 (12.3)	< 0.001
II	367 (14.9)	216 (21.9)	
III	807 (32.8)	355 (36.0)	
IV	1098 (44.6)	293 (29.7)	
Chemotherapy, yes, n (%)	1439 (58.4)	544 (55.2)	0.091
Radiotherapy, yes, n (%)	150 (6.1)	82 (8.3)	0.022
Surgery, yes, n (%)	475 (19.3)	266 (27.0)	< 0.001
CRP (mean (SD))	20.78 (22.79)	1.52 (0.92)	< 0.001
Albumin (mean (SD))	37.19 (8.37)	42.48 (15.63)	< 0.001
TBIL (mean (SD))	14.27 (22.66)	12.48 (11.35)	0.018
AST (mean (SD))	30.15 (42.49)	26.42 (24.29)	0.010
ALT (mean (SD))	26.76 (34.2)	25.62 (28.28)	0.357
WBC, 10^9^/L, mean (SD)	6.99 (3.82)	5.76 (3.24)	
Neutrophil, 10^9^/L, mean (SD)	5.49 (7.54)	3.59 (4.32)	< 0.001
Lymphocyte, 10^9^/L, mean (SD)	1.65 (2.75)	1.56 (1.89)	0.353
RBC (mean (SD))	4.13 (2.28)	4.39 (3.15)	0.007
Platelet (mean (SD))	242.96 (104.15)	211.98 (85.11)	< 0.001
KPS (mean (SD))	81.63 (15.06)	86.83 (11.60)	< 0.001
PG-SGA, mean (SD)	9.01 (4.99)	6.65 (4.54)	< 0.001
EORTC QLQ-C30, mean (SD)	52.65 (11.12)	49.36 (9.45)	< 0.001

Data are represented as mean (SD), median (interquartile range), or number (%)

*IQR* Interquartile range, *BMI* Body mass index, *CRP* C-reactive protein, *TBIL* Total bilirubin, *AST* Alanine aminotransferase, *ALT* Aspartate aminotransferase, *WBC* White blood cell, *RBC* Red blood count, *KPS* Karnofsky Performance Status, *PG-SGA* Patient-generated subjective nutrition assessment, *EORTC QLQ-C30* European Organization for Research and Treatment of Cancer Quality of Life Questionnaire

For NCR, low < 309.07; high ≥ 309.07

### Subgroup analysis of modifiers affecting NCR and OS relationship

**Fig. 4:**
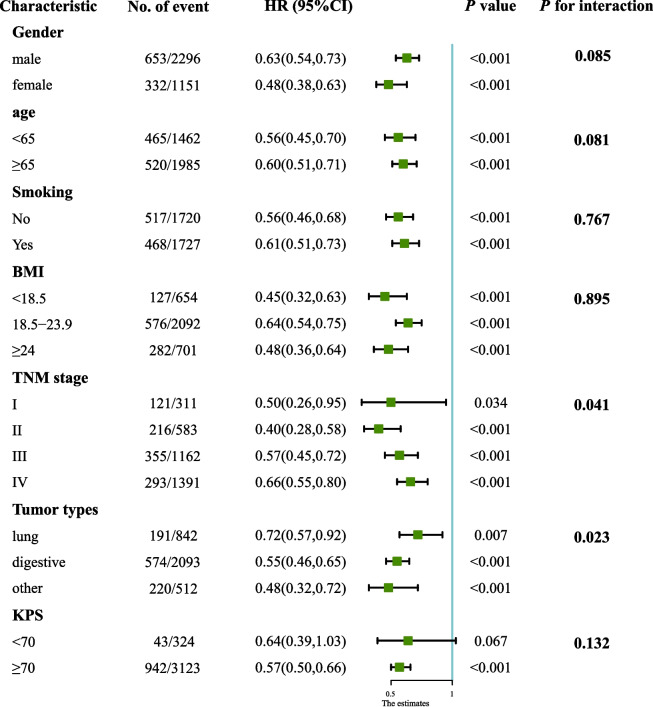
The association between NCR (stratified by cut-offs 309.07) and hazard ratios of overall survival in various subgroups**.** Except the stratifying variable, the model is adjusted by sex, age, smoking, tumor type, TNM stage, chemotherapy, surgery, albumin, TBIL, neutrophil count, platelet count, KPS score, PG-SGA score and EORTC QLQ-C30 score. KPS, Karnofsky Performance Status Scale; PG-SGA, patient-generated subjective nutrition assessment; EORTC QLQ-C30, European Organization for Research and Treatment of Cancer Quality of Life Questionnaire; CI, confidence interval; HR, hazard ratio

The subgroup analysis revealed insights into the relationship between NCR and OS across various patient characteristics and tumor types. For Fig. 4, the analysis demonstrated that higher NCR was consistently associated with improved survival outcomes across different subgroups, including gender, age, smoking status, BMI, TNM stage, and tumor types. Specifically, both males and females with higher NCR exhibited better OS, with HR indicating a significantly lower risk of death (HR for males: 0.63, 95% CI: 0.54–0.73; HR for females: 0.48, 95% CI: 0.38–0.63). This trend was consistent across age groups, smoking status, various BMI categories, tumor types, and TNM stages. However, this association was not found among patients with a KPS < 70. Further analysis focused on survival rates correlated with NCR levels, stratified by tumor class and TNM stage. As observed from the Kaplan–Meier (KM) curves (Supplementary Fig. 5), cachexia patients with low NCR had the worst survival outcomes when they were in an advanced TNM stage and had lung cancer (all log-rank *p* < 0.001).

### Sensitivity analysis

Given the association between NCR and the prognosis of patients with cancer cachexia, we conducted a sensitivity analysis to ensure the stability of our research findings by excluding those who died within 6 months of follow-up or had severe comorbidities (Table 3). When considering NCR continuously, the HR for mortality risk showed a significant decrease with each SD increase in NCR, both when excluding patients dying within 6 months (HR 0.80, 95% CI: 0.74–0.86, *p* < 0.001) and those without severe comorbidities (HR 0.79, 95% CI: 0.73–0.85, *p* < 0.001). When categorizing NCR by its cutoff value (309.07), a high NCR was associated with a significantly lower risk of mortality compared to a low NCR, with HRs of 0.57 (95% CI: 0.50–0.65, *p* < 0.001) for the group excluding early deaths and 0.54 (95% CI: 0.47–0.62, *p* < 0.001) for patients without severe comorbidities. Interquartile range analysis further detailed the relationship between NCR levels and mortality risk. Compared to the first quartile (Q1), the third quartile (Q3) and fourth quartile (Q4) demonstrated a significant reduction in mortality risk, particularly notable for Q3 without severe comorbidities (HR 0.52, 95% CI: 0.44–0.61, *p* < 0.001) and Q4 in both analysis scenarios.

**Table 3:** Hazard risk for all-cause mortality in cachexia patients with low NCR by excluding patients dying within 6 months or patients with severe comorbidities

NCR	HR 95%CI	*p*-value	HR 95%CI	*p*-value
Sensitive analysis	Excluding patients dying within 6 months		Without severe comorbidities	
As continuous (per SD)	0.80 (0.74,0.86)	< 0.001	0.79 (0.73,0.85)	< 0.001
By NCR cut-off				
Low (< 309.07)	Ref		Ref	
High (≥ 309.07)	0.57 (0.50,0.65)	< 0.001	0.54 (0.47,0.62)	< 0.001
Interquartile				
Q1 (< 39.49)	Ref		Ref	
Q2 (39.49–155.93)	1.01 (0.88,1.16)	0.915	0.99 (0.86,1.14)	0.858
Q3 (155.93–341.76)	0.86 (0.74,0.99)	0.047	0.52 (0.44,0.61)	0.004
Q4 (≥ 341.76)	0.56 (0.48,0.66)	< 0.001	0.81 (0.77,0.86)	< 0.001
*p* for trend		< 0.001		< 0.001

Adjusted by age, sex, TNM stage, tumor type, surgery, radiotherapy, chemotherapy, hypertension, diabetes, smoking, drinking, family history

## Discussion

In this study, we evaluated the prognostic value of a novel inflammatory and nutritional index—the NCR (Nutritional CRP Ratio)—in patients with cancer cachexia. The NCR is calculated using the formula: BMI (kg/m^2^) × albumin (g/L) / C-reactive protein (mg/L), aiming to simultaneously reflect the patient's nutritional status and level of inflammation. Our findings suggest that the NCR, as a comprehensive indicator, can effectively predict the survival prognosis of patients with cancer cachexia. Lower NCR levels were associated with higher all-cause mortality rates across various types of cancer cachexia, indicating that malnutrition and systemic inflammation are key factors in the progression of cancer cachexia. Additionally, the study identified 309.07 as the optimal NCR threshold; patients with values above this threshold exhibited better OS. This emphasizes the importance of assessing and monitoring patients clinically using the NCR.

Inflammation not only catalyzes the onset and advancement of tumors but is also a hallmark of cancer cachexia [1, 17]. Cancer-associated inflammation includes both local and systemic inflammation, with these two forms interacting in the development and progression of cancer, significantly impacting patient survival outcomes. Local inflammation typically manifests as immune responses within the tumor microenvironment, promoting tumor cell proliferation, migration, and drug resistance, while systemic inflammation exacerbates immune suppression throughout the body by releasing various inflammatory factors, such as TNF-α and IL-6, further promoting tumor metastasis and recurrence [18–20]. C-reactive protein (CRP), as a marker of systemic inflammation, holds substantial value in determining the prognosis of cancer patients [21]. Building on this, other important indices based on CRP, such as the Lymphocyte/CRP ratio (LCR), CRP × neutrophil/lymphocyte ratio (IBI), and CRP/albumin ratio (CAR), have demonstrated even greater prognostic value than CRP alone [8, 22]. Similarly, in our study, we combined CRP with nutritional indicators such as BMI and albumin to create NCR, which has been shown to outperform CRP alone in predicting the prognosis of cancer cachexia. Compared to other biomarkers, such as NLR and PLR, which primarily focus on either inflammation or nutritional status, NCR integrates both aspects, making it a more comprehensive and reliable prognostic tool. By incorporating both nutritional status and systemic inflammation, NCR offers a more holistic approach to assessing cancer cachexia, addressing the critical factors in cancer progression and potentially providing superior prognostic value in certain clinical settings.

Patients experiencing malnutrition tend to have a poorer response to cancer treatments and face an increased risk of mortality associated with cachexia [23]. The malnutrition observed in patients with cancer cachexia is not solely a result of reduced food intake but also stems from metabolic changes induced by both the tumor and the host's inflammatory response to the tumor [24]. Tumor cells activate systemic inflammatory responses by secreting various pro-inflammatory cytokines [25]. These cytokines not only directly affect the metabolism of nutrients, leading to the breakdown of fat and muscle, but also suppress appetite and alter neuroendocrine functions by reducing food intake, ultimately resulting in weight loss and muscle mass reduction [26]. Simultaneously, nutrition regulates inflammation and immune responses by affecting metabolism, microbial composition, and endocrine factors, thereby influencing disease progression and treatment responses [27]. Thus, it is crucial to concurrently assess the nutritional status and systemic inflammation levels in patients with cancer cachexia. This approach enhances the precision of prognostic predictions and offers a more holistic evaluation of the condition. This aligns with the recommendations of the ESMO guidelines, which emphasize malnutrition and systemic inflammation as the two core factors for diagnosing cancer cachexia [28]. In our research, we integrated markers of inflammation and nutritional status. Through multivariate survival analyses, adjusted by various models and supplemented with sensitivity analyses, we demonstrated that the NCR holds significant predictive value for survival in patients with cachexia. NCR can also serve as an independent prognostic factor for overall survival, aiding in risk stratification of colorectal cancer patients, particularly those in UICC stages II and III. It helps optimize personalized treatment strategies for colorectal cancer, especially in selecting patients who are most likely to benefit from adjuvant therapy, demonstrating significant clinical value.

Studies have revealed a profound connection between systemic inflammation and the experiences of fatigue or a reduction in subjective functioning [29, 30]. Additionally, malnutrition significantly deteriorates the quality of life in cancer patients by compromising physical functionality and diminishing survival capabilities [31]. Our research supported these insights, indicating that low NCR is associated with a decline in function and may correlate with a deterioration in the quality of life among patients with cancer cachexia. Furthermore, malnutrition and systemic inflammation tend to be more prevalent in advanced stages of cancer [32], a trend that is echoed in the findings of our analysis. Our findings also reveal that most types of cancer exhibit low NCR status in cachexia, with the notable exceptions of gastric, pancreatic, and endometrial cancers, suggesting a broad applicability of NCR as a marker in the context of cancer cachexia.

This study has several limitations. The assessment of NCR was performed at only a single time point, without continuous monitoring of NCR for each patient throughout the follow-up period. The decision to use a single baseline NCR value was made due to its clinical relevance as an initial prognostic marker at diagnosis, providing important information for early risk stratification and treatment decisions. Continuous monitoring of NCR changes over time would be challenging due to treatment effects, confounding factors, and practical difficulties in repeated measurements. Future studies may explore the potential value of monitoring NCR dynamics, but for the scope of this research, baseline NCR proved to be a sufficient and reliable indicator for prognosis. Furthermore, the use of maximally selected rank statistics to determine the NCR cut-off value does have inherent limitations, including the potential for overfitting, inflation of Type I error due to multiple testing, and a lack of sufficient clinical rationale. The cut-off value determined in this study may need further validation in independent cohorts and clinical settings. Future research will focus on this aspect to ensure the generalizability and clinical applicability of the NCR cut-off value.

## Conclusion

This study validates the NCR as a crucial prognostic tool for cancer cachexia, providing a new perspective on assessing nutritional status and systemic inflammation. The utilization of a dual-marker approach illuminates the interconnected roles of malnutrition and inflammation in the advancement of cancer, while also facilitating the development of personalized therapeutic strategies to address these significant factors. Through the identification of an optimal NCR threshold, our research underlines the importance of early detection and intervention, potentially revolutionizing the clinical care and prognostication of individuals with cancer cachexia.

## Supplementary Information

Supplementary Material 1: Figure 1. Comparison of the value of NCR and its individual indicators in predicting the prognosis of patients with cancer cachexia using AUCs curves. The x-axis denotes the overall survival time, and the y-axis signifies the estimated area under the ROC curve for survival at the specified time. BMI, body mass index; CRP, C-reactive protein.

Supplementary Material 2: Figure 2. NCR (log transformation) levels of patients with cancer cachexia in different TNM stages.

Supplementary Material 3: Figure 3. Associations between the NCR and clinical parameters. KPS, Karnofsky Performance Status Scale; PG-SGA, Patients-generated subjective nutritional assessment; EORTC QLQ-C30, European Organization for Research and Treatment of Cancer Quality of Life Questionnaire.

Supplementary Material 4: Figure 4. Overall survival in patients with cancer cachexia based on the NCR cut-off. For NCR low <309.07, high ≥309.07.

Supplementary Material 5: Figure 5. KM curves of OS for patients stratified by low and high NCR and covariates with interactions. *p*-values were computed by the log-rank test. NCR low <309.07, high ≥309.07.

Supplementary Material 6: Table 1. Univariate and multivariate Cox regression analysis of factors associated with overall survival. BMI, body mass index; CRP, C-reactive protein; TBIL, total bilirubin; AST, alanine aminotransferase; ALT, aspartate aminotransferase; WBC, white blood cell; RBC, red blood count; KPS, Karnofsky Performance Status; PG-SGA, patient-generated subjective nutrition assessment; EORTC QLQ-C30, European Organization for Research and Treatment of Cancer Quality of Life Questionnaire. Table 2. Hazard risk for special cancer overall survival in cachexia patients with high NCR. Hazard risk is adjusted by age, sex, TNM stage, tumor type, surgery, radiotherapy, chemotherapy, hypertension, diabetes, smoking, drinking, family history, except for the stratifying variable. Table 3. Quality of life stratified by cut-off point of NCR. Data are represented as median (interquartile range).

## Data Availability

No datasets were generated or analysed during the current study.
