# Supplementary figures and images for: NCR as a biomarker for nutritional status and inflammation in predicting outcomes in patients with cancer cachexia: a prospective, multicenter study
Source: BMC Cancer. 2025 Mar 25;25:539. doi: 10.1186/s12885-025-13919-1 (PMC11934689; doi:10.1186/s12885-025-13919-1)

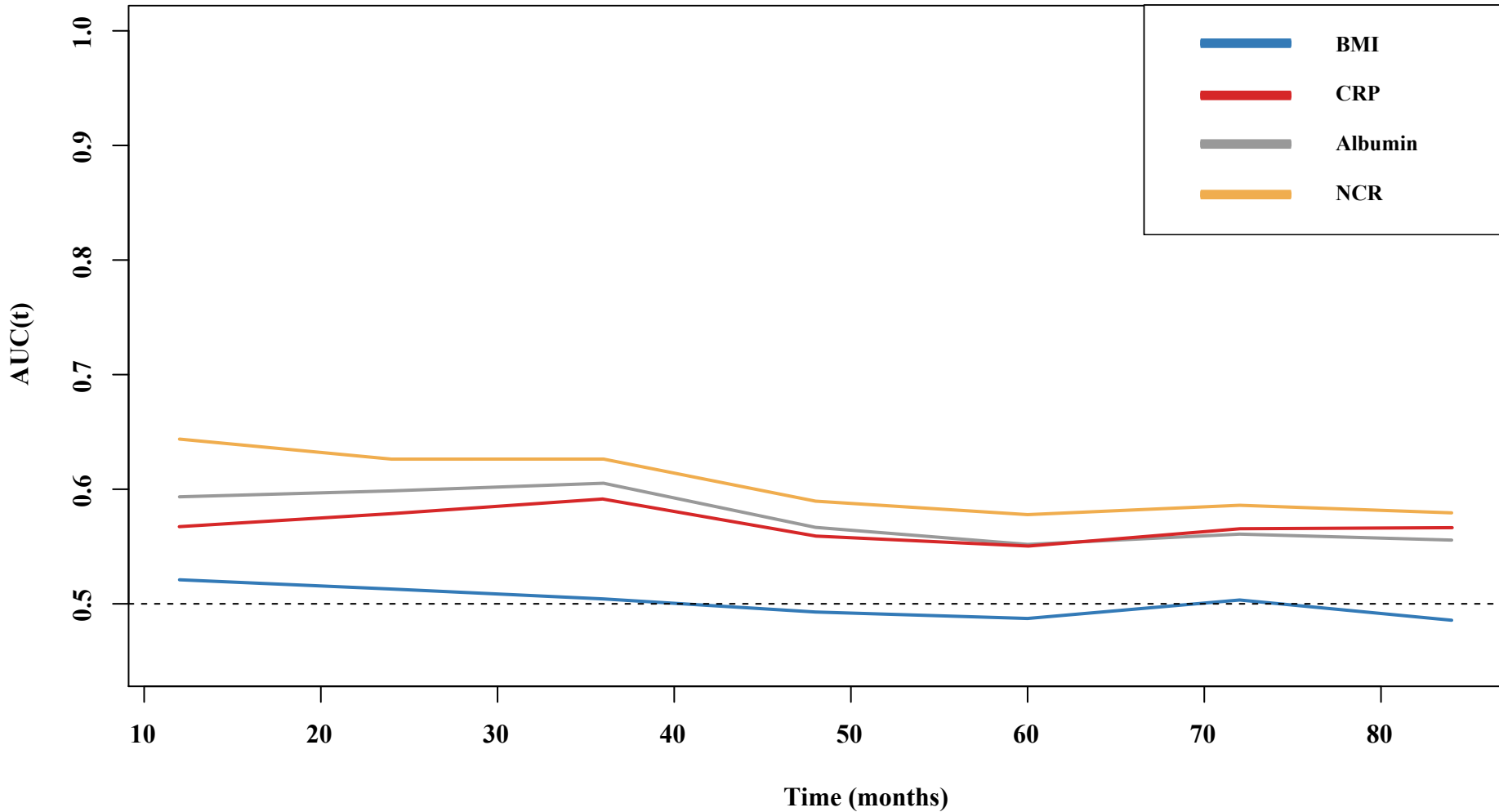

Supplement: Supplementary file 1 — Supplementary Material 1: Figure 1. Comparison of the value of NCR and its individual indicators in predicting the prognosis of patients with cancer cachexia using AUCs curves. The x-axis denotes the overall survival time, and the y-axis signifies the estimated area under the ROC curve for survival at the specified time. BMI, body mass index; CRP, C-reactive protein. [file 12885_2025_13919_MOESM1_ESM.pdf]

logNCR

3

2

1

I

II

III

IV

TNM stage

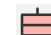

I

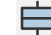

II

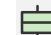

III

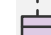

IV

\*\*\*

\*\*\*

\*\*\*

\*\*\*

Supplement: Supplementary file 2 — Supplementary Material 2: Figure 2. NCR (log transformation) levels of patients with cancer cachexia in different TNM stages. [file 12885_2025_13919_MOESM2_ESM.pdf]

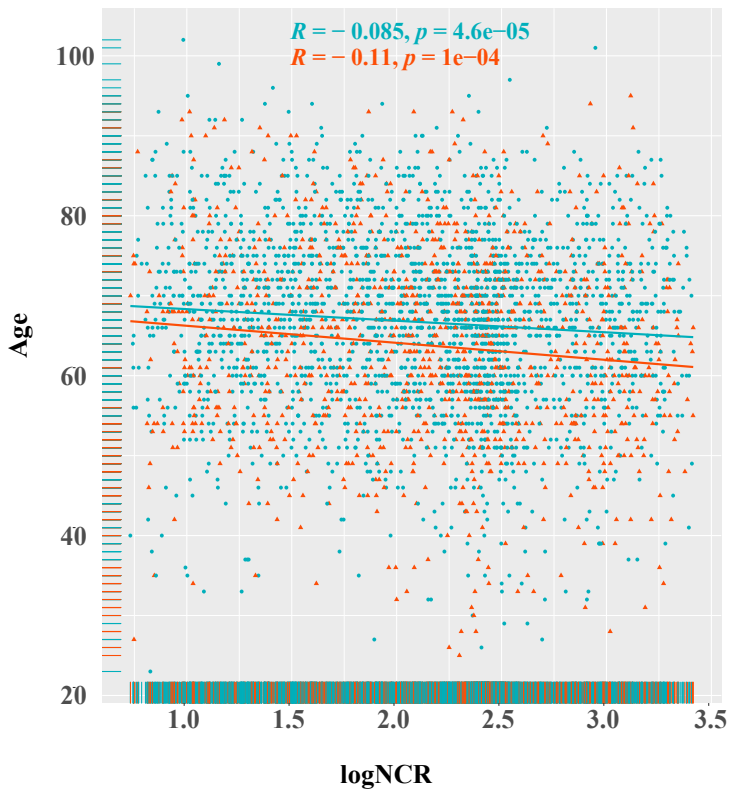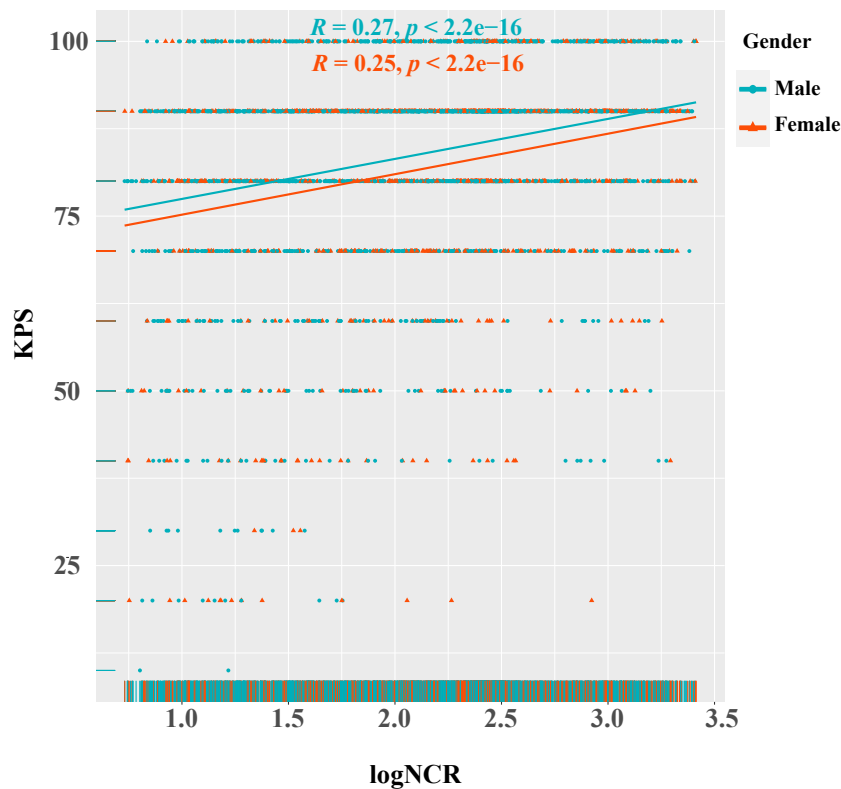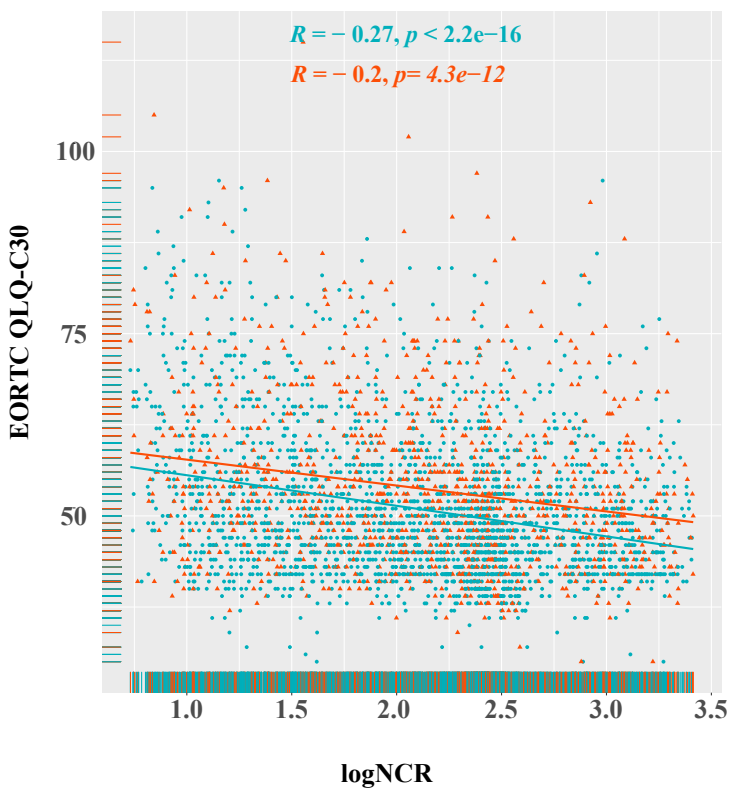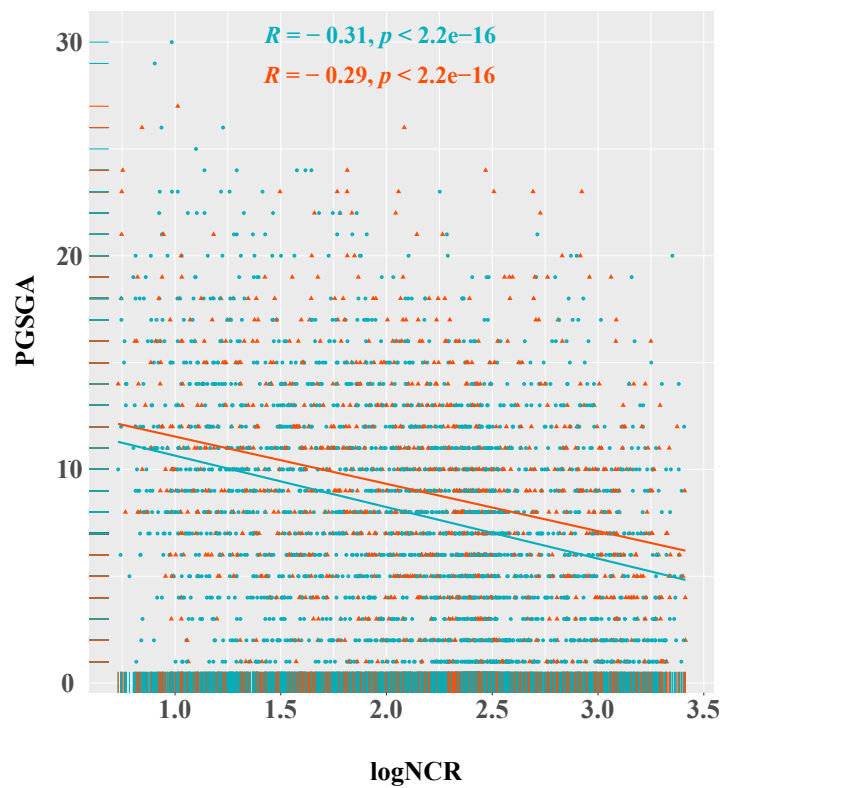

Supplement: Supplementary file 3 — Supplementary Material 3: Figure 3. Associations between the NCR and clinical parameters. KPS, Karnofsky Performance Status Scale; PG-SGA, Patients-generated subjective nutritional assessment; EORTC QLQ-C30, European Organization for Research and Treatment of Cancer Quality of Life Questionnaire. [file 12885_2025_13919_MOESM3_ESM.pdf]

A

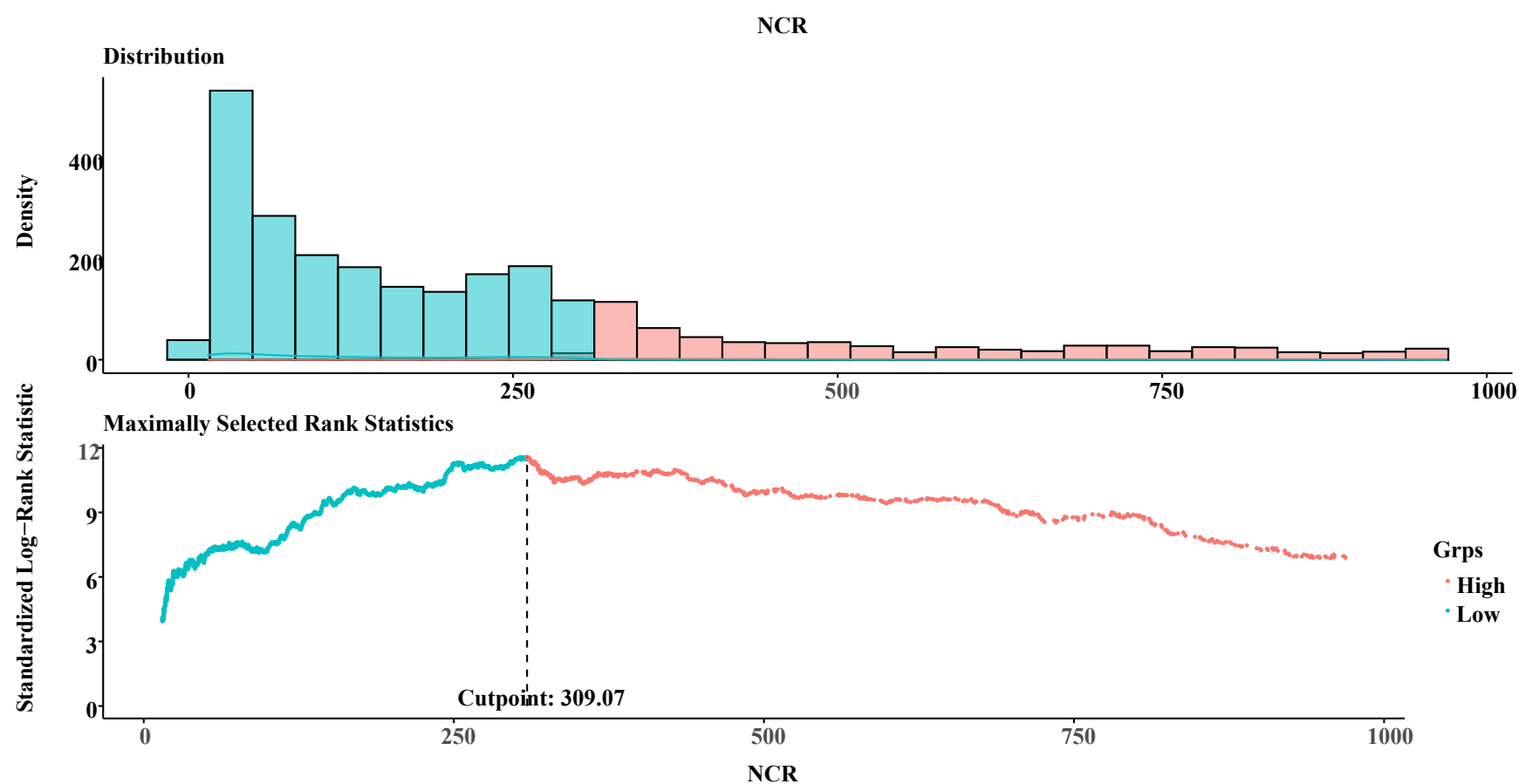

B

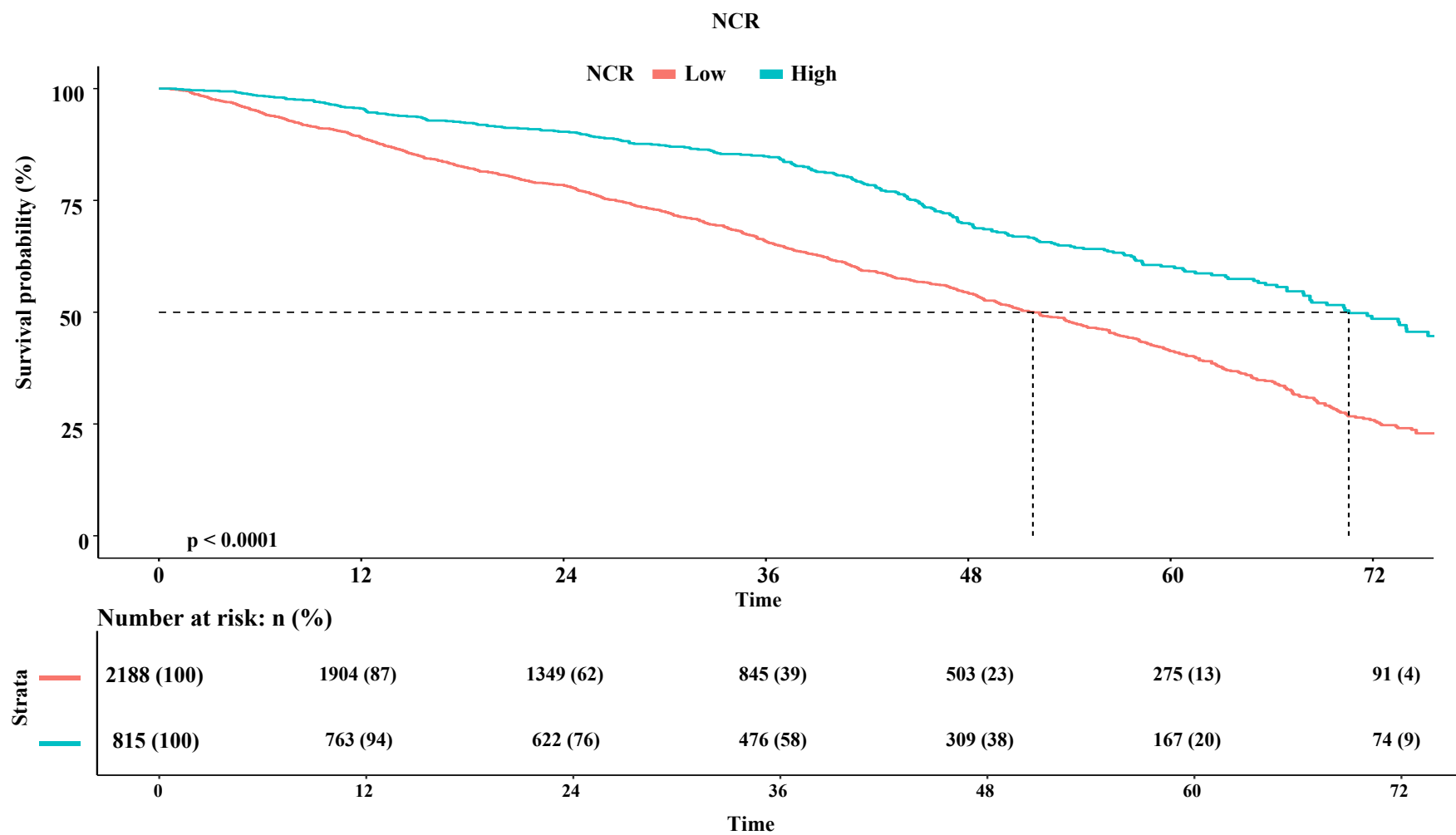

Supplement: Supplementary file 4 — Supplementary Material 4: Figure 4. Overall survival in patients with cancer cachexia based on the NCR cut-off. For NCR low <309.07, high ≥309.07. [file 12885_2025_13919_MOESM4_ESM.pdf]

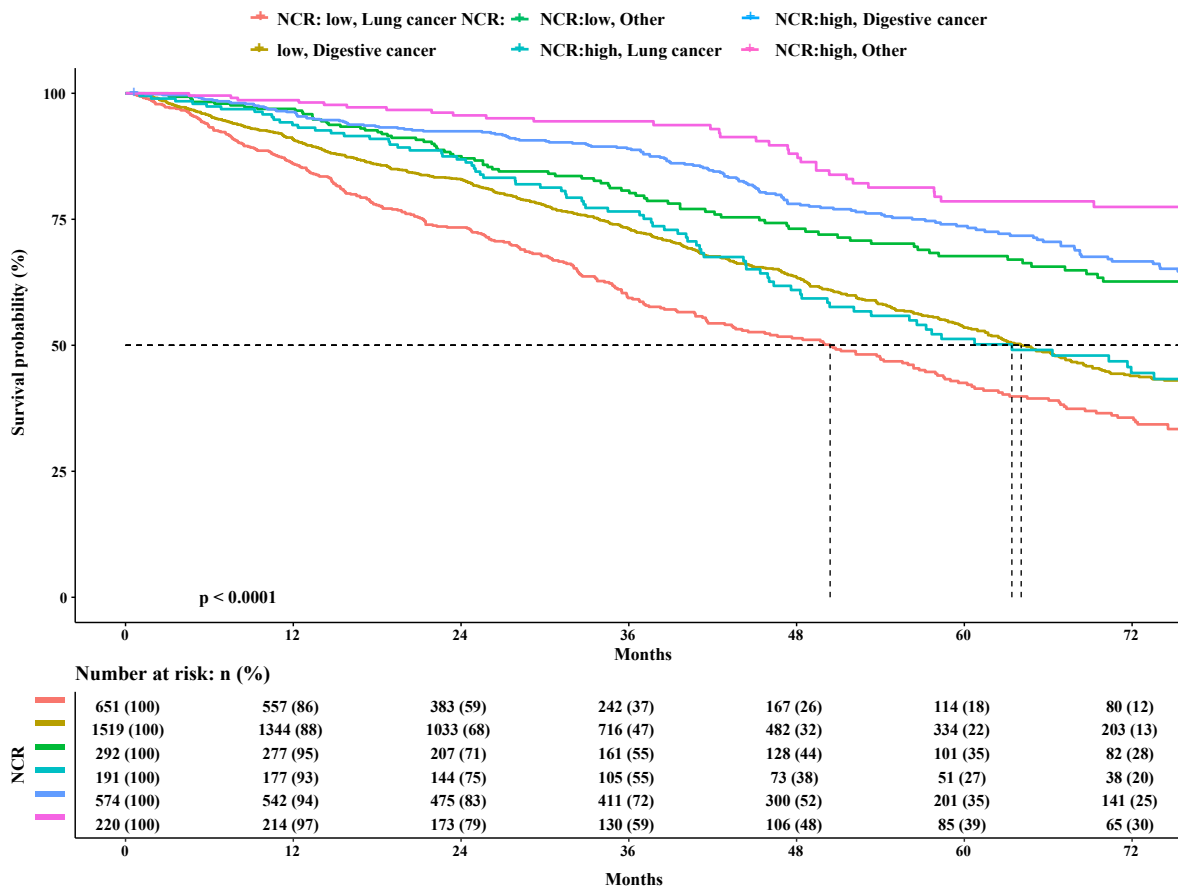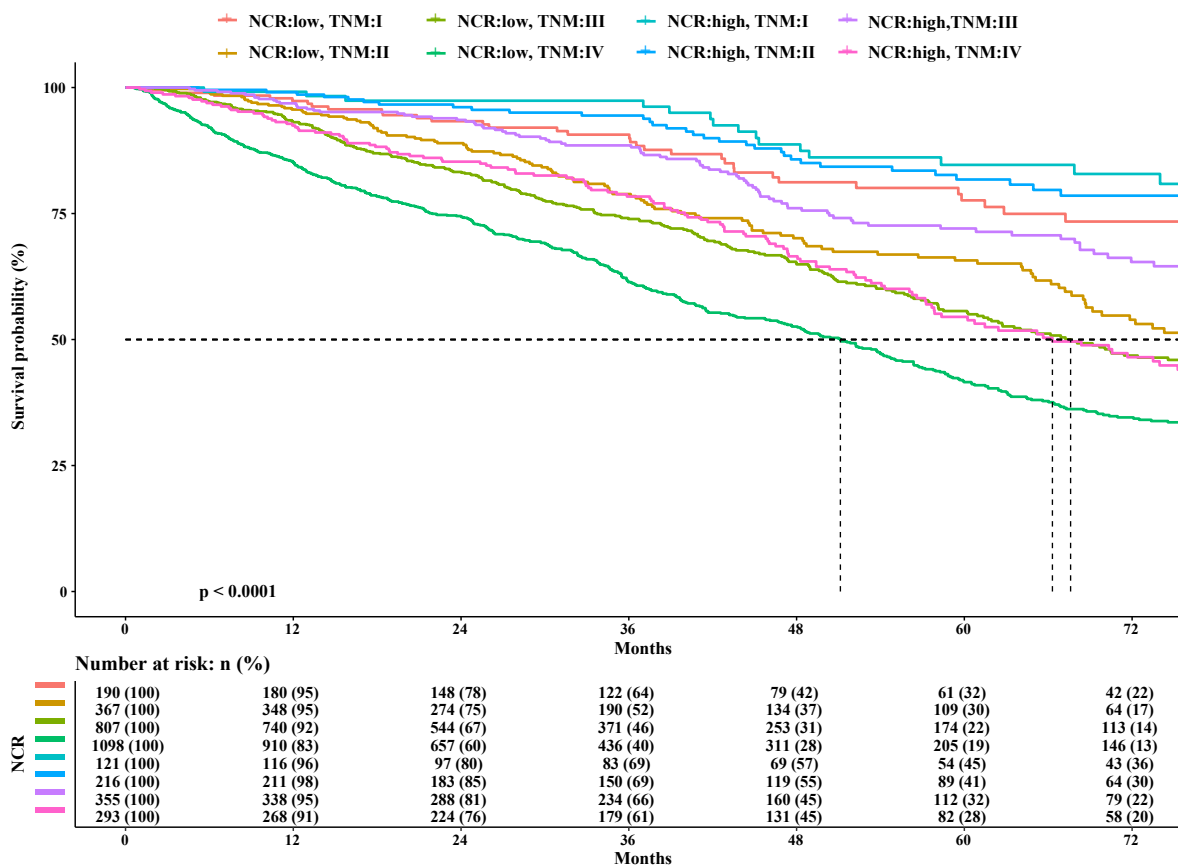

Supplement: Supplementary file 5 — Supplementary Material 5: Figure 5. KM curves of OS for patients stratified by low and high NCR and covariates with interactions. p-values were computed by the log-rank test. NCR low <309.07, high ≥309.07. [file 12885_2025_13919_MOESM5_ESM.pdf]
