# Supplementary material for: NCR as a biomarker for nutritional status and inflammation in predicting outcomes in patients with cancer cachexia: a prospective, multicenter study
Source: BMC Cancer. 2025 Mar 25;25:539. doi: 10.1186/s12885-025-13919-1 (PMC11934689; doi:10.1186/s12885-025-13919-1)
Supplement: Supplementary file 6 — Supplementary Material 6: Table 1. Univariate and multivariate Cox regression analysis of factors associated with overall survival. BMI, body mass index; CRP, C-reactive protein; TBIL, total bilirubin; AST, alanine aminotransferase; ALT, aspartate aminotransferase; WBC, white blood cell; RBC, red blood count; KPS, Karnofsky Performance Status; PG-SGA, patient-generated subjective nutrition assessment; EORTC QLQ-C30, European Organization for Research and Treatment of Cancer Quality of Life Questionnaire. Table 2. Hazard risk for special cancer overall survival in cachexia patients with high NCR. Hazard risk is adjusted by age, sex, TNM stage, tumor type, surgery, radiotherapy, chemotherapy, hypertension, diabetes, smoking, drinking, family history, except for the stratifying variable. Table 3. Quality of life stratified by cut-off point of NCR. Data are represented as median (interquartile range). [file 12885_2025_13919_MOESM6_ESM.docx]

Supplementary Table 1. Univariate and multivariate Cox regression analysis of factors associated with overall survival.

| Characteristic | Univariate |  | Multivariate | |
| --- | --- | --- | --- | --- |
|  | HR 95% CI | *p*-Value | HR 95% CI | *p*-Value |
| Sex | 0.75 (0.67,0.84) | <0.001 | 0.90 (0.77,1.04) | 0.155 |
| Age | 1.01 (1.01,1.02) | <0.001 | 1.00 (1.00,1.01) | 0.370 |
| BMI | 0.99 (0.97,1.00) | 0.096 |  |  |
| Diabetes | 1.40 (1.19,1.65) | 0.052 |  |  |
| Hypertension | 1.18 (1.04,1.34) | 0.012 | 1.07 (0.94,1.22) | 0.313 |
| Smoke | 1.33 (1.20,1.48) | <0.001 | 1.21 (1.06,1.38) | 0.005 |
| Alcohol | 1.29 (1.15,1.45) | 0.138 |  |  |
| Tea | 1.07 (0.95,1.21) | 0.273 |  |  |
| Clinical Characteristic |  |  |  |  |
| Lung cancer | 1.77 (1.58,1.99) | <0.001 | 1.22 (1.01,1.47) | 0.039 |
| liver cancer | 1.17 (0.88,1.57) | 0.282 |  |  |
| Gastric cancer | 0.88 (0.78,0.99) | 0.034 | 0.99 (0.81,1.20) | 0.039 |
| Colorectal cancer | 0.70 (0.60,0.81) | <0.001 | 0.71 (0.57,0.87) | 0.001 |
| Esophagus cancer | 1.18 (0.97,1.42) | 0.094 |  |  |
| Pancreatic cancer | 2.58 (2.03,3.29) | <0.001 | 1.81 (1.35,2.41) | <0.001 |
| Gynecological and breast cancer | 0.33 (0.25,0.43) | <0.001 | 0.57 (0.42,0.78) | 0.001 |
| Other cancer | 0.67 (0.54,0.83) | <0.001 | 0.56 (0.43,0.73) | <0.001 |
| TNM stage |  |  |  |  |
| I |  |  |  |  |
| II | 1.41 (1.06,1.88) | 0.017 | 1.40 (1.05,1.86) | 0.022 |
| III | 2.10 (1.62,2.72) | <0.001 | 1.94 (1.49,2.53) | <0.001 |
| IV | 3.33 (2.59,4.29) | <0.001 | 2.65 (2.04,3.44) | <0.001 |
| Radiotherapy | 0.81 (0.64,1.01) | 0.060 |  |  |
| Chemotherapy | 1.22 (1.10,1.36) | <0.001 | 1.17 (1.03,1.33) | 0.016 |
| Surgery | 0.63 (0.55,0.72) | <0.001 | 0.86 (0.72,1.03) | 0.112 |
| Albumin | 0.98 (0.97,0.99) | 0.001 |  |  |
| CRP | 1.01 (1.01,1.01) | <0.001 |  |  |
| NCR | 0.66 (0.61,0.71) | <0.001 | 0.80 (0.73,0.87) | <0.001 |
| TBIL | 1.01 (1.00,1.01) | <0.001 | 1.00 (1.00,1.01) | 0.020 |
| WBC | 1.02 (1.01,1.03) | 0.025 | 1.00 (0.98,1.01) | 0.903 |
| Neutrophil | 1.01 (1.00,1.01) | 0.553 |  |  |
| Lymphocyte | 0.99 (0.97,1.02) | 0.546 |  |  |
| AST | 1.00 (1.00,1.00) | <0.001 | 1.00 (1.00,1.00) | 0.046 |
| ALT | 1.00 (1.00,1.00) | <0.001 | 1.00 (1.00,1.00) | 0.543 |
| RBC | 0.99 (0.97,1.02) | 0.611 |  |  |
| Platelet | 1.00 (1.00,1.00) | <0.001 | 1.00 (1.00,1.00) | 0.015 |
| KPS | 0.99 (0.98,0.99) | <0.001 | 1.00 (0.99,1.00) | 0.341 |
| PG-SGA | 1.04 (1.03,1.05) | <0.001 | 1.02 (1.00,1.03) | 0.008 |
| EORTC QLQ-C30 | 1.38 (1.22,1.55) | <0.001 | 1.06 (0.92,1.22) | 0.454 |

BMI, body mass index; CRP, C-reactive protein; TBIL, total bilirubin; AST, alanine aminotransferase; ALT, aspartate aminotransferase; WBC, white blood cell; RBC, red blood count; KPS, Karnofsky Performance Status; PG-SGA, patient-generated subjective nutrition assessment; EORTC QLQ-C30, European Organization for Research and Treatment of Cancer Quality of Life Questionnaire.

Supplementary Table 2. Hazard risk for special cancer overall survival in cachexia patients with high NCR.

| Tumor types | HR 95%CI | *p*-value |
| --- | --- | --- |
| Lung cancer | 0.75 (0.58,0.95) | 0.020 |
| liver cancer | 1.30 (0.58,2.93) | 0.520 |
| Gastric cancer | 0.66 (0.51,0.86) | 0.002 |
| Colorectal cancer | 0.45 (0.31,0.65) | <0.001 |
| Esophagus cancer | 0.45 (0.25,0.84) | 0.012 |
| Pancreatic cancer | 0.86 (0.40,1.86) | 0.708 |
| Gynecological and breast cancer | 0.30 (0.14,0.67) | 0.003 |
| Other cancer | 0.88 (0.53,1.45) | 0.614 |

Hazard risk is adjusted by age, sex, TNM stage, tumor type, surgery, radiotherapy, chemotherapy, hypertension, diabetes, smoking, drinking, family history, except for the stratifying variable.

Supplementary Table 3. Quality of life stratified by cut-off point of NCR.

| Characteristic | NCR Low | NCR High | *p*-value |
| --- | --- | --- | --- |
|  | n=2,462 | n=985 |  |
| Physical function | 86.67 (26.67) | 86.67 (20.00) | <0.001 |
| Role function | 66.67 (33.33) | 83.33 (33.33) | <0.001 |
| Emotional function | 91.67 (25.00) | 91.67 (25.00) | 0.103 |
| Cognitive function | 100.00 (16.67) | 100.00 (16.67) | <0.001 |
| Social function | 66.67 (4.38) | 66.67 (33.33) | <0.001 |
| Global QOL | 66.67 (16.67) | 66.67 (33.33) | <0.001 |
| Fatigue | 22.22 (33.33) | 11.11 (33.33) | <0.001 |
| Nausea and vomiting | 0.00 (0.00) | 0.00 (0.00) | <0.001 |
| Pain | 0.00 (33.33) | 0.00 (16.67) | <0.001 |
| Dyspnea | 0.00 (33.33) | 0.00 (0.00) | <0.001 |
| Sleep disturbance | 0.00 (33.33) | 33.33 (33.33) | <0.001 |
| Appetite loss | 0.00 (33.33) | 0.00 (33.33) | <0.001 |
| Constipation | 0.00 (0.00) | 0.00 (0.00) | 0.089 |
| Diarrhea | 0.00 (0.00) | 0.00 (0.00) | 0.321 |
| Financial difficulties | 33.33 (33.33) | 33.33 (33.33) | <0.001 |

Data are represented as median (interquartile range).
